# Siropins, novel serine protease inhibitors from gut microbiota acting on human proteases involved in inflammatory bowel diseases

**DOI:** 10.1186/s12934-016-0596-2

**Published:** 2016-11-29

**Authors:** Héla Mkaouar, Nizar Akermi, Vincent Mariaule, Samira Boudebbouze, Nadia Gaci, Florette Szukala, Nicolas Pons, Josan Marquez, Ali Gargouri, Emmanuelle Maguin, Moez Rhimi

**Affiliations:** 1 UMR 1319 Micalis, INRA, AgroParisTech, Université Paris-Saclay, 78350 Jouy-en-Josas, France; 2 Laboratory of Molecular Biology of Eukaryotes, Center of Biotechnology of Sfax, University of Sfax, 3038 Sfax, Tunisia; 3 European Molecular Biology Laboratory, Grenoble Outstation, 71 Avenue des Martyrs, CS 90181, 38042 Cedex 9 Grenoble, France; 4 INRA, Institut National de la Recherche Agronomique, US 1367 Metagenopolis, Jouy-en-Josas, France

**Keywords:** Commensal bacteria, Serpin, Fecal protease, Biochemical studies

## Abstract

**Background:**

In eukaryotes, the serpins constitute a wide family of protease inhibitors regulating many physiological pathways. Many reports stressed the key role of serpins in several human physiopathologies including mainly the inflammatory bowel diseases. In this context, eukaryotic serpins were largely studied and their use to limit inflammation was reported. In comparison to that, bacterial serpins and mainly those from human gut microbiota remain poorly studied.

**Results:**

The two genes encoding for putative serpins from the human gut bacterium *Eubacterium sireaum*, display low sequence identities. These genes were overexpressed and the encoded proteins, named Siropins, were purified. Activity studies demonstrated that both purified proteins inhibited serine proteases but surprisingly they preferentially inhibited two human serine proteases (Human Neutrophil Elastase and Proteinase3). The biochemical characterization of these Siropins revealed that Siropin 1 was the most active and stable at low pH values while Siropin 2 was more thermoactive and thermostable. Kinetic analysis allowed the determination of the stoichiometry of inhibition (SI) which was around 1 and of the association rate constants of 7.7 × 10^4^ for the Human Neutrophil Elastase and 2.6 × 10^5^ for the Proteinase3. Moreover, both Siropins displayed the ability to inhibit proteases usually present in fecal waters. Altogether our data indicate the high efficiency of Siropins and their probable involvement in the control of the overall intestine protease activity.

**Conclusions:**

Here we report the purification and the biochemical characterization of two novel serpins originated from *Eubacterium sireaum*, a human gastro-intestinal tract commensal bacteria. These proteins that we called Siropins, efficiently inhibited two human proteases reported to be associated with inflammatory bowel diseases. The determination of the biochemical properties of these enzymes revealed different temperature and pH behaviours that may reflect adaptation of this human commensal bacterium to different ecological environments. To the best of our knowledge, it is the first bacterial serpins showing an attractive inhibition of fecal proteases recovered from a mice group with chemically induced inflammation. Altogether our data highlight the interesting potential of Siropins, and serpins from the human gut microbiota in general, to be used as new alternative to face inflammatory diseases.

**Electronic supplementary material:**

The online version of this article (doi:10.1186/s12934-016-0596-2) contains supplementary material, which is available to authorized users.

## Background

The serine protease inhibitors (serpins) represent the most widely distributed superfamily of protease inhibitors having been identified across all branches of life including eukaryotes as well as viruses and prokaryotes [1, 2]. Serpins are being extensively studied in humans and they have been reported to inhibit proteolytic activities involved in many physiological processes including: inflammatory responses, blood coagulation and fibrinolysis [3, 4]. The viral serpins are associated to virulence through the inactivation of the proteases of the host immune system [5, 6]. Moreover, serpins also operate through non inhibitory mechanisms on many physiological functions such as tumor suppression, molecular chaperone activity, chromatin compaction and hormone transport [1]. Unlike small protease inhibitors acting by a reversible way, serpins are distinguishable by their irreversible suicide mechanism of inhibition [7]. Indeed, the serpin inhibition activity is associated to the Reactive Center Loop (RCL) which interacts with target protease thereby resulting in the cleavage of the RCL that enables the establishment of a covalent acyl-enzyme complex. These main structural reshuffles will induce an important distortion of the protease leading to its inactivation [8].

Comparatively to the knowledge on the eukaryotic serpins function, that regarding the bacterial serpins is still very limited [1]. In fact, until now only few microbial serpins were reported including those from *Clostridium thermocellum, Tannerella forsythia, Thermobifida fusca* and *Thermoanaerobacter tengcondensis* [9–13]. While these bacterial serpins were characterized, their physiological roles are still to be investigated.

Interestingly, only one serpin from the human commensal bacterium *Bifidobacterium longum* NCC2705 strain was studied. In fact, it was demonstrated that this bacterial serpin inhibited several eukaryotic serine proteases and mainly the Human Neutrophil Elastase (HNE) [14]. Taking into account that eukaryotic serine proteases are associated with several human protease-mediated physiopathologies and essentially inflammatory bowel diseases (IBD), the serpins can constitute a promising therapeutic approach to treat such diseases [14]. This claim is strengthened by the demonstration that the human specific inhibitor of HNE (Elafin) allowed the reduction of induced digestive inflammation in a rodent model [15]. In this framework, the interest towards the bacterial serpins from the human gut microbiota is taking more importance when one considers the higher numbers of these polypeptides compared to the 36 serpins encoded by the human body [1]. The recent scientific breakthroughs in the gut microbiota studies clearly demonstrated the association between the gut microbiota and IBD [16, 17]. Consequently, the serpins encoded by the human gut microbiota today appear as attractive candidates to counteract the deleterious damages associated with the GIT-derived protease activities and may ensure an important competitive advantage to survive in this ecological context [14, 18]. Therefore, serpins from the gut microbiota may have therapeutic potential which remains hitherto unexplored.

Here we report the cloning, over-expression, purification and biochemical characterization of two novel serpins isolated from the human commensal *Eubacterium sireaum*. The purified serpins were characterized. They display differential inhibitor efficiency against human serine proteases. Interestingly, these proteins constitute the first bacterial serpins inhibiting the human Proteinase3 and the fecal proteases from inflamed mice.

## Results and discussion

### Genes in silico analysis, cloning and over-expression

**Fig. 1:**
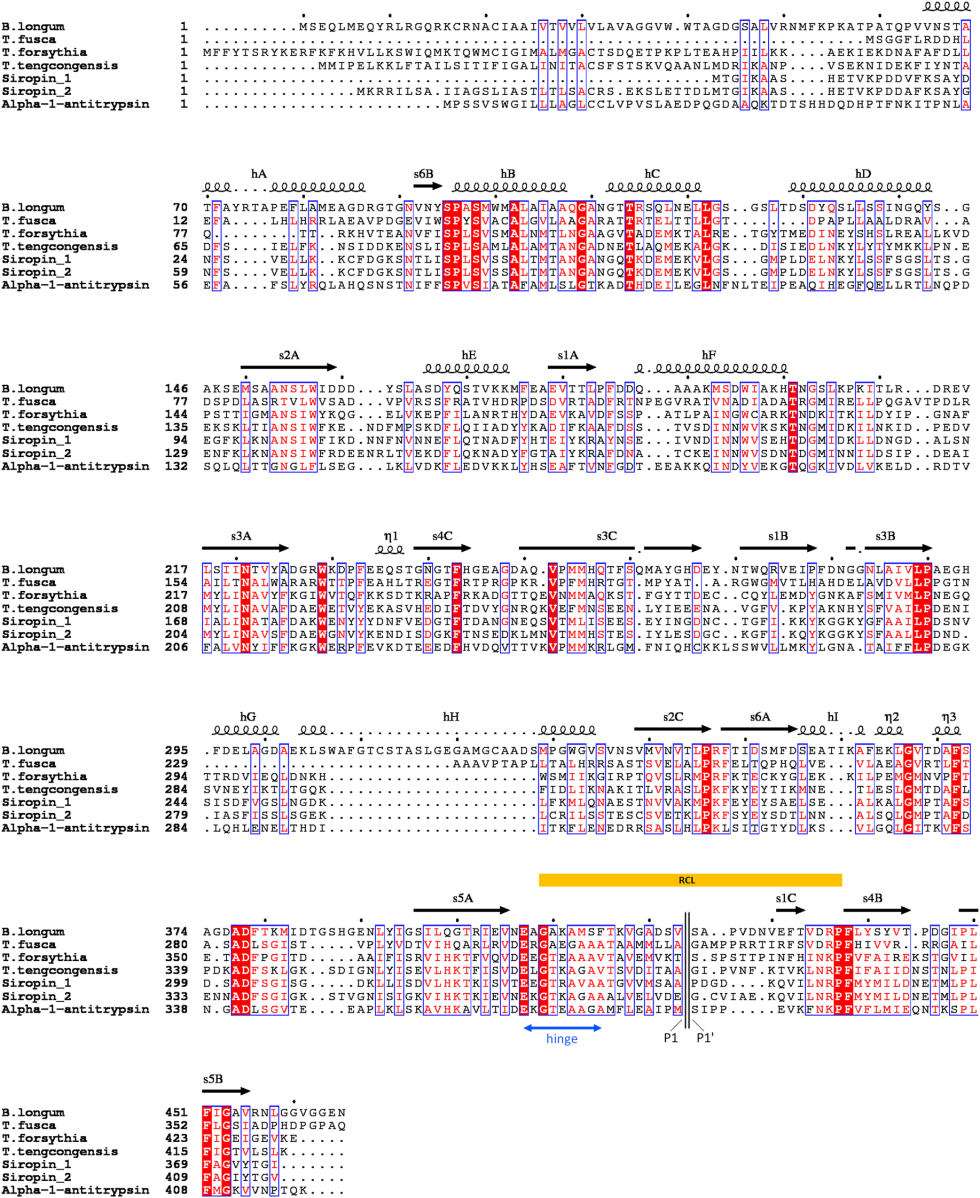
Multiple sequence alignment of Siropin 1 and 2 with Thermopin (Uniprot accession number Q47NK3R), Miropin (Uniprot accession number G8UQY8), human α-1-antitrypsin (UniProt accession number P01009), *B. longum* serpin (UniProt accession number Q8G7X7) and Tengpin (UniProt accession number Q8R9P5). The structural elements shown above the alignment were generated using the native α-1-antitrypsin structure (PDB ID: 1QLP) sequence invariant residues between sequences are typed *red* on a *white background* and residues conserved within each group are displayed as *white letters* on a *red background*. *Underlined sequences* represent the predicted hinge region (*blue*) and reactive center loop (*yellow*). The predicted cleavage site is marked with a double vertical line. P1 and P1′ residues are labeled

Analysis of the amino-acid sequence multiple alignment demonstrates that Siropin 1 and 2 have 60% of identity. Sequence inspection of the Siropin 1 and 2 with other serpins reveals that they display sequence identities of only 19 and 20% with the previously studied serpin from the gut bacterium *B. longum* (Fig. 1). The same study revealed that the serpins from *Tannerella forsythia* and *Thermobifida fusca* displayed low identities of 23 and 24% with the Siropin 1 and of 21 and 19% with Siropin 2. By using TMHMM and SignalP programs we conclude that Siropin 1 is an intracellular protein; but Siropin 2 was significantly predicted as an extracellular protein (data not shown). The sequence identity of the two Siropins increased to 63% when the presumed signal sequence was omitted from Siropin 2. In addition, the alignment of the RCL sequences from Siropins displays an identity value of only 48%. These low sequence identities can be explained by what was previously suggested concerning the serpin genes in prokaryotes i.e., that they are most probably prone to horizontal gene transfer [2, 19].

As shown in Fig. 1, both Siropins possess three β sheets, eight α helices and an exposed RCL which are the structural elements that characterize all members of serpin family [2, 20]. Moreover, analysis of the RCL region demonstrates that the Siropins hinge sequences are mainly composed by small and highly conserved amino acids including Glycine and Alanine (Fig. 1). This observation suggests that the two proteins studied herein most probably act as proteases inhibitors [2, 21].

**Fig. 2:**
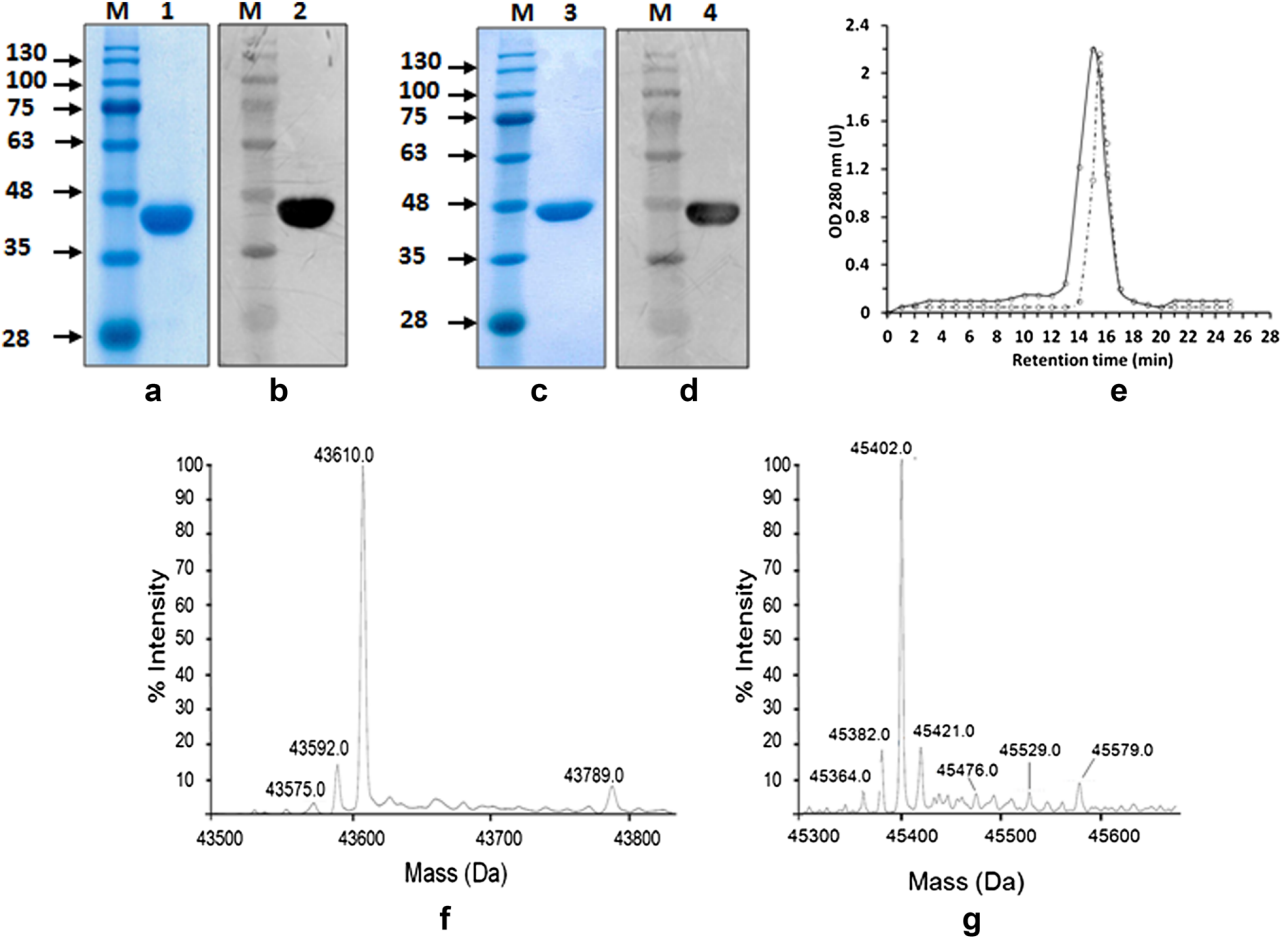
Electrophoretic, size exclusion chromatography and mass spectrometry analysis of the purified Siropins. **a** and **c** SDS-PAGE of purified Siropin 1 and Siropin 2, respectively. **b** and **d** Western blot detection of purified Siropin 1 and Siropin 2, respectively. *Lane M* protein marker (molecular mass in kilodaltons); *lane 1* and *2* purified Siropin 1; *lane 3* and *4* purified Siropin 2. **e** Size exclusion chromatography analysis of the purified recombinant proteins shows a single peak of 50 kDa for Siropin 2 (RT 15 min, *continue line*) and 45 kDa for Siropin 1 (RT 15.5 min, *dashed line*) using protein markers of 669 kDa (TR 8.9 min), 440 kDa (TR 11.3 min), 158 kDa (TR 13.2 min), 75 kDa (TR 14.1 min), 44 kDa (TR 15.3 min) and 29 kDa (TR 16.9 min). **f** and **g** Mass spectrometry analysis of the Siropin 1 and Siropin 2, respectively

To investigate this hypothesis, two DNA fragments of approximately 1.5 kb were amplified using *Eubacterium siraeum* chromosomal DNA as template and two oligonucleotides designed for each gene. These DNA fragments were cloned under control of the T7 promoter and in frame with six histidine residues at the N-terminal side of the encoded proteins. For both Siropins, structural model showed that the N-termini are located away from the active sites (Additional file 1: Fig. S1). The calculated molecular weights were 43.75 and 48.1 kDa for Siropin 1 and Siropin 2, respectively. After transformation into *E. coli* BL21 (DE3), numerous colonies were observed and subsequently analyzed by PCR and DNA sequencing. Monitoring of the liquid culture of each selected clone followed by western blotting using the intracellular crude extract, showed the presence of bands with a molecular weight of nearly 44 and 46 kDa close to the theoretically expected one (Fig. 2b, d). These results were confirmed by mass spectrometry analysis which proved the correspondence of these protein species to Siropin 1 and 2 (data not shown). These data demonstrate the molecular cloning and the expression of the *E. siraeum* serpin genes in *E. coli* BL21.

### Purification of Siropins, spectrum and stoichiometry of inhibition

After an overnight liquid cell cultures of the recombinant *E. coli* BL21 strains over-expressing both serpins, the protein crude extracts were subjected to an affinity chromatography step. High purity enzyme preparations were obtained with size exclusion chromatographies which displayed single elution peaks with apparent molecular masses of 45 kDa and 50 kDa for Siropin 1 and 2, respectively (Fig. 1e). The electrophoresis under reducing conditions and mass spectrometry analysis revealed the presence of homogenous single bands with molecular masses of around 44 and 46 kDa (Fig. 2a, c, f and g), suggesting that both serpins from *E. siraeum* have a monomeric arrangement. Such organization is similar to those adopted by serpins from *Thermococcus kodakaraensis*, *Tannerella forsythia, Pyrobaculum aerophilum* and *Thermobifida fusca* [10, 12, 22, 23].

**Fig. 3:**
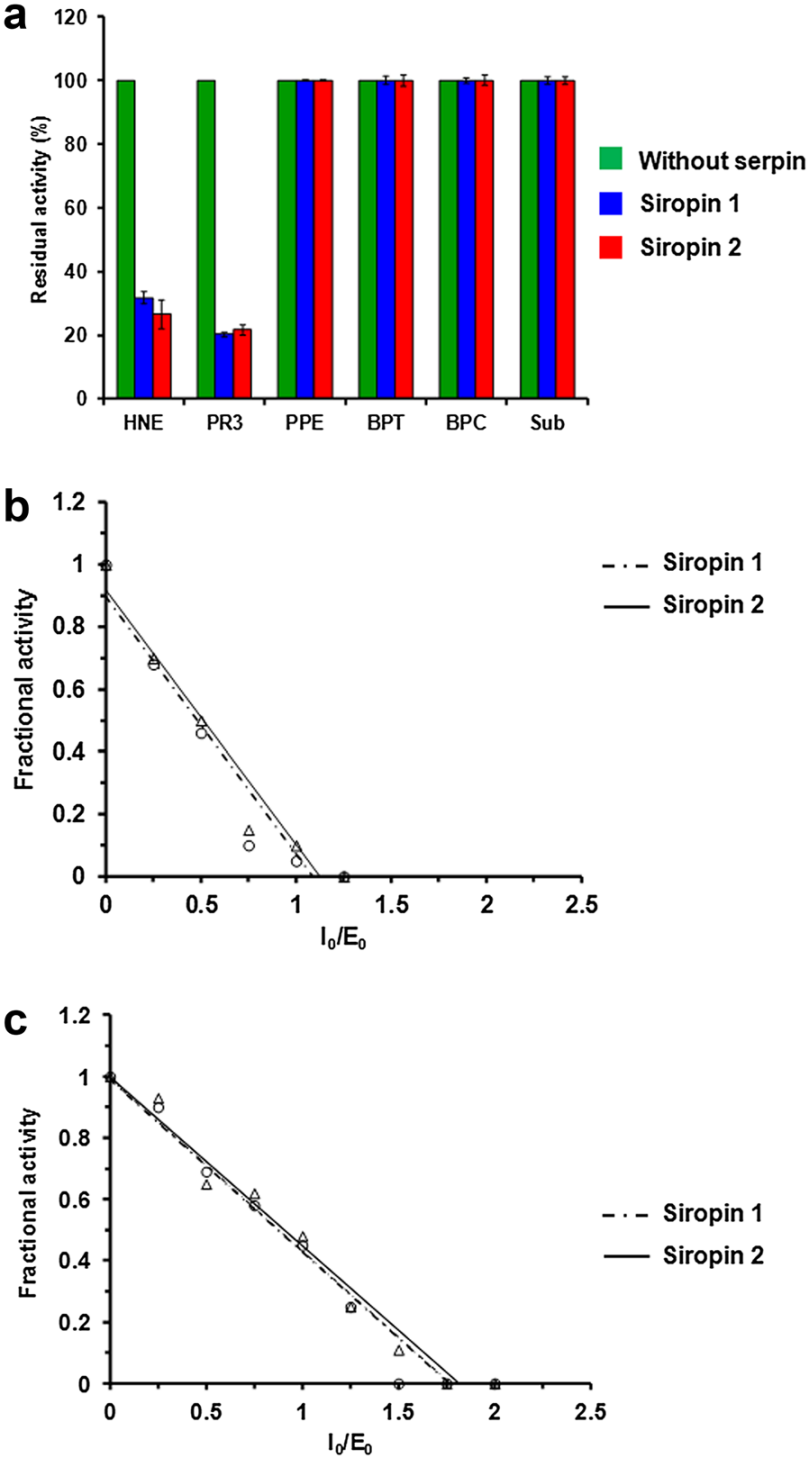
Spectrum and stoichiometry of inhibition of purified Siropins. **a** Effect of Siropin1 and 2 on *HNE* human neutropil elastase, *PR3* proteinase 3, *PPE* porcine pancreatic elastase, *BPT* bovine pancreatic trypsine, *BPC* bovine pancreatic chymotrypsine and *Sub* subtilisine. The activity of each protease was assayed alone (*green*), pre-incubated with Siropin 1 (*blue*) or with siropin 2 (*red*). Measured protease activities without serpins were defined as 100%. *Error bars* represent the standard deviation from three independent experiments. **b** and **c** Stoichiometry of inhibition of Siropins. Fractional protease activity of HNE (**b**) and PR3 (**c**) was plotted after incubation with various amounts of Siropin 1 (*Dashed*, ^_^o^_^) or Siropin 2 (*Continuous*, ^_^∆^_^)

Interestingly, purification yields were about 12 mg and 8 mg of protein per liter of culture for Siropin 1 and 2, respectively. In comparison, previously reported purification yields per liter were 25 µg, 3 and 10 mg for serpins from *Tannerella forsythia*, *Thermococcus kodakaraensis* and *Bifidobacterium longum* [10, 14, 22]. Altogether these data indicate that the Siropin 1 and 2 are well expressed in *E. coli* and that the used purification procedure allowed the achievement of high yields of serpins. The obtained pure protein amounts were sufficient for the investigation of the inhibition spectrum of Siropins. As shown in Fig. 3a, none of the Siropins inhibited the pancreatic porcine elastase (PPE), bovine pancreatic trypsin (BPT) and chymotrypsin (BPC). The same result was also observed when using subtilisin (Sub), the bacterial serine protease from *Bacillus subtilis*. In contrast, Siropin 1 and 2 efficiently inhibited two human serine proteases: the Human Neutrophil elastase (HNE) and the Proteinase3 (PR3). It is noteworthy that with previously reported serine proteases even at 10 or 20 fold higher molar concentrations, no inhibition was observed.

In the case of HNE and PR3 the inhibition level was dependent upon Siropins concentration; it allowed us to determine the stoichiometry of inhibition (SI). Figure 3b, c revealed that Siropin 1 has SI values of 1.76 and 1.1 for HNE and PR3, respectively. Siropin 2 shows SIs of 1.82 for HNE and 1.1 for PR3 (Fig. 3b, c). SI values of Siropins with HNE are lower than that of serpin from *Tannerella forsythia* (SI = 3.4) and similar to those of serpins from *B. longum*, *T. tengcondensis* and of the human α-1-antitrypsin [13, 14, 24]. Concerning the PR3, the SIs of Siropins were similar to that of the human α-1-antitrypsin [25]. Furthermore as far as we know, Siropins constitute the first bacterial serpins inhibiting the human PR3. It is well recognized that serpins allow the defense of bacteria through the inhibition of proteolytic activities contributing thus to their adaptation towards ecological environment. At least, it was reported that the serpins from *Bacillus brevis* and *Bifidobacterium breve* protected these bacteria from exogenous attacks through the inhibition of proteases [18, 26]. In the case of *E. sireaum*, our data reavealed that it can use its Siropins to ensure a protection against the host proteases, HNE and PR3. Similar behavior was previously described in case of the ecotin from *E. coli* [27].

Above all it appeared that Siropins act efficiently against human proteases while they were not active against the *B. subtilis* subtilisin. This feature takes more importance when one considers that (i) HNE and PR3 are highly over-expressed in subjects suffering from inflammatory bowel diseases (IBD) when compared to healthy one [15, 28, 29] and (ii) many reports stressed the involvement of serine proteases on human inflammatory physiopathology’s and mainly HNE and PR3 [15, 28, 29].

### Identification of the RCL cleavage sites

**Fig. 4:**
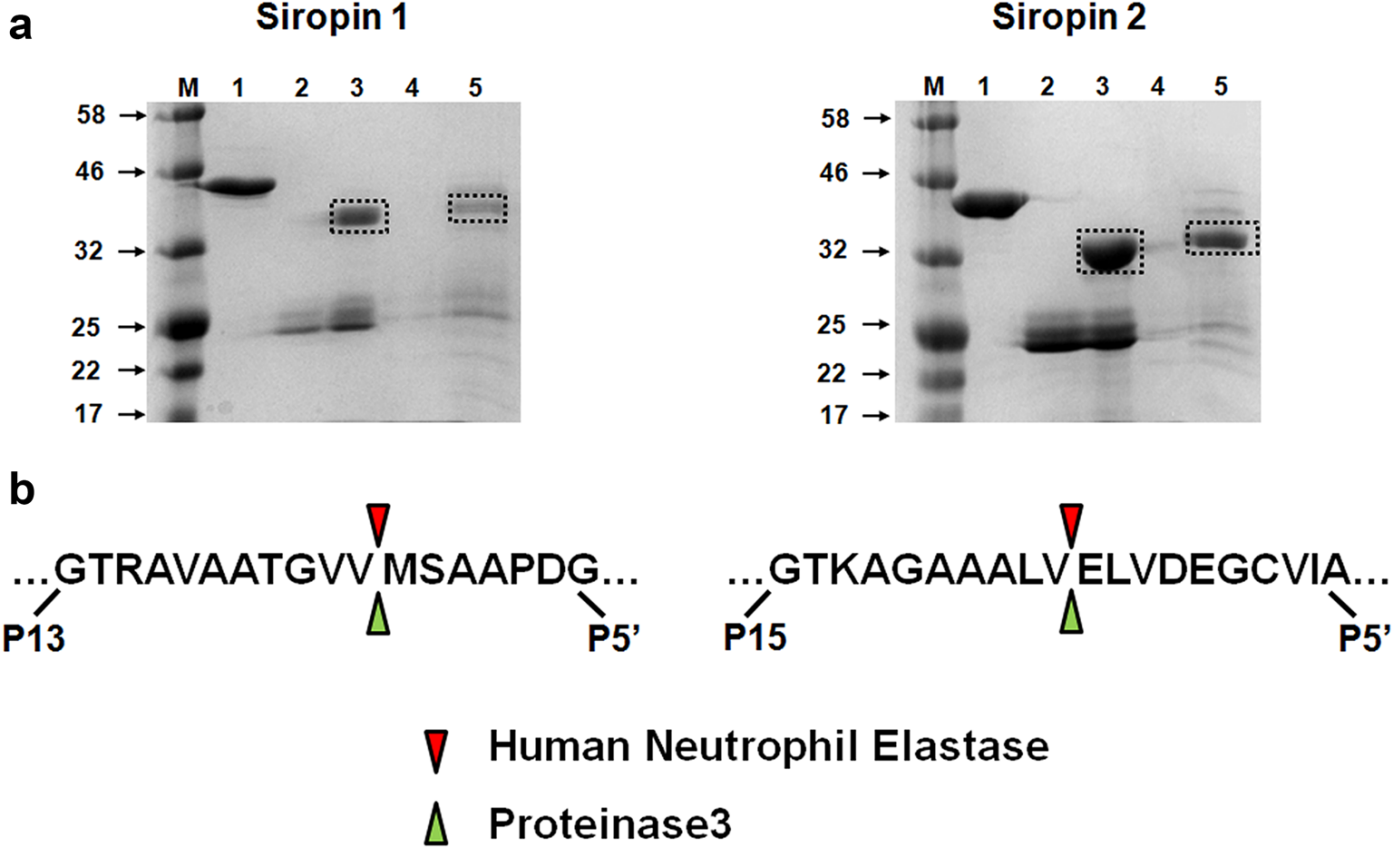
Determination of the RCL cleavage sites of the Siropin 1 and 2 using mass spectrometry analysis. **a** The stained protein bands labelled with frames were excised and subjected to mass spectrometry analysis. Siropin 1: *M* protein marker; *1* Siropin 1; *2* HNE; *3* Siropin 1 + HNE; *4* PR3; *5* Siropin 1 + PR3. Siropin 2: *M* protein marker; *1* Siropin 2; *2* HNE; *3* Siropin 2 + HNE; *4* PR3; *5* Siropin 2 + PR3. **b** Diagram of the reactive center loops showing the cleavage sites for each Siropin identified with HNE and PR3

In order to determine the P1–P1′ cleavage site in the RCL of Siropins, the purified proteins were incubated with target proteases and subsequently analyzed by SDS-PAGE and mass spectrometry (Fig. 4). In the case of Siropins, the complex band was not detectable by SDS-PAGE analysis. This kind of complex instability has been previously reported with other inhibitory serpins and it is assumed that it results from a nucleophile or water attack that can occur upon the complex formation leading to the release of cleaved serpin and distorted protease [30]. In both case (HNE and PR3) the intensity of the band corresponding to untreated serpin (44 for Siropin 1 and 46 kDa for Siropin 2) decreased, while a slightly lower band appear upon addition of the target protease (Fig. 4a). Further mass spectrometry analyses were performed on the digestion band and obtained results revealed that this band correspond to the RCL-cleaved Siropins (data not shown). Subsequently, based on the sequence analysis of this cleaved form, we successfully determined the Siropins cleavage sites which correspond to P3-P2 (Val338-Met339) peptide bonds for Siropin 1 and P6-P5 (Val375-Glu376) bond for Siropin 2 both for HNE and PR3 (Fig. 4b). Interestingly, those cleavage sites are consistent with the specificity of HNE and PR3 for Val or Ala at P1 position.

### Effects of temperature and pH on Siropins activity and stability

**Fig. 5:**
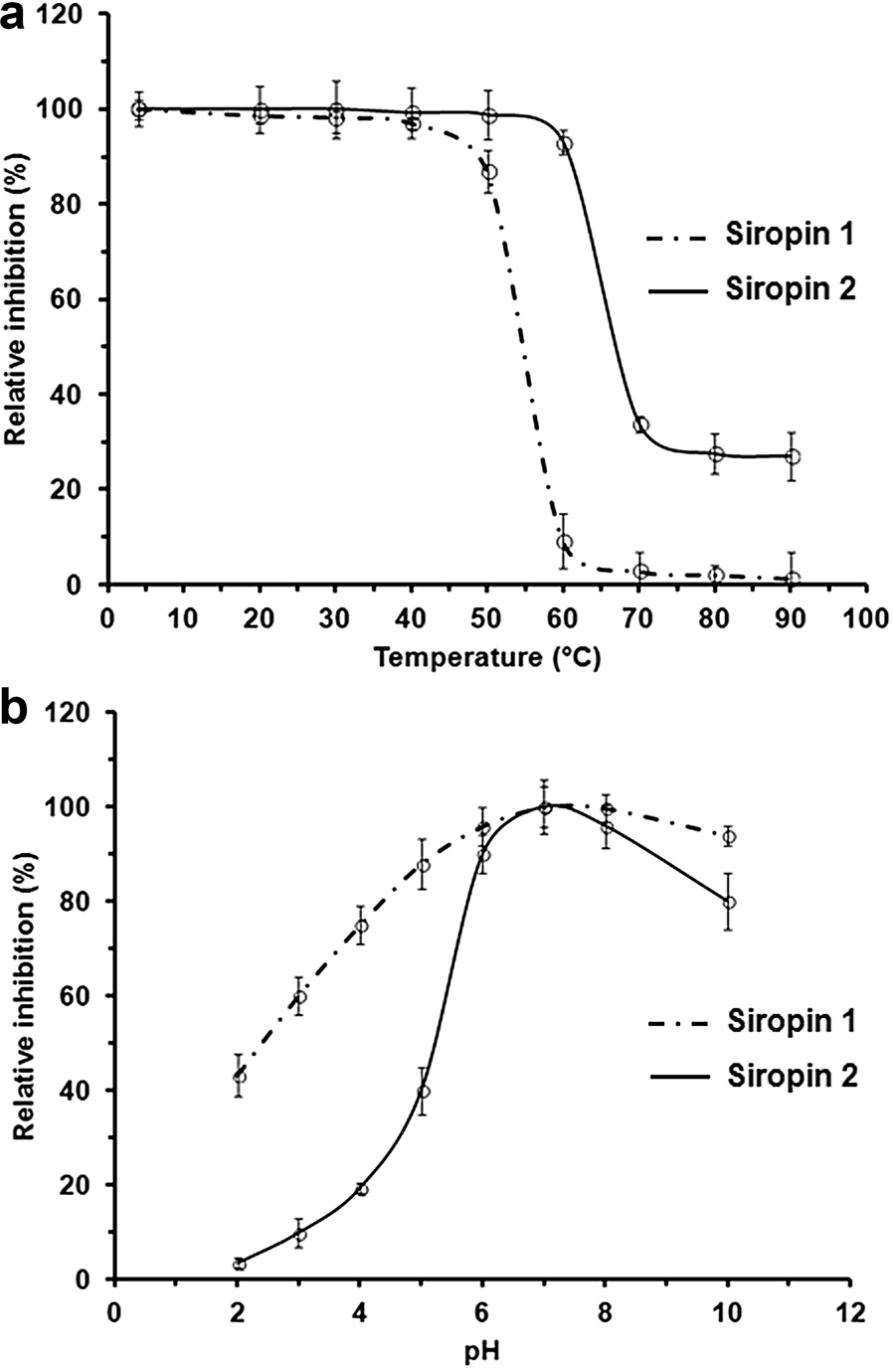
Effect of temperature (**a**) and pH (**b**) on the inhibitory effect of the purified Siropin 1 (*dashed line*) and Siropin 2 (*continuous line*). Inhibitions at the optimal pH and the optimal temperature were defined as 100%. *Error bars* represent the standard deviation from three independent experiments

The analysis of the inhibition patterns at different temperatures demonstrated that Siropin 2 was fully active at temperatures until 50 °C (Fig. 5a). The same study established that Siropin 1 was fully active up to 40 °C and displayed a relative inhibition of 88% at 50 °C. Amazingly, at 60 °C Siropin 2 preserves more than 90% of its activity whereas Siropin 1 kept less than 10% of its activity. At temperatures above 60 °C, Siropin 2 retained 30-35% of its initial activity but Siropin 1 was almost inactive (Fig. 5a). Therefore, we conclude that both Siropins are highly active at low temperatures, while at high temperatures the Siropin 2 is more thermoactive than Siropin 1. In comparison the previously described bacterial serpin from a marine uncultured microorganism and *Thermococcus kodakaraensis* displayed optimal inhibitions of 25 and 100 °C, respectively [22, 31].

Study of pH effect on Siropins revealed that these proteins were highly active at pH 7.0 (Fig. 5b). Moreover, they retained more than 75% of their activities from pH 6.0 to 10. Remarkably, Siropin 1 displayed higher relative activities at pH values ranged between 2.0 and 5.0. For example at pH 3.0, the relative activities of Siropin 1 and Siropin 2 were 60 and 10% respectively. Under high pH values, Siropin 1 also kept a higher relative activity than Siropin 2 e.g., at pH 10 Siropin 1 and 2 preserved 97 and 80% of their relative activities, respectively.

Previous studies of the serpin deriving from a marine uncultured microorganism showed an optimal pH range of 7.0 to 8.0 and an optimal pH of 7.0 for the serpin isolated from *Haemonchus contortus* [31, 32].

Our results established that Siropin 2 is distinguishable from Siropin 1 by its tolerance to high temperatures while Siropin 1 was active at extreme pH values compared to Siropin 2. In the other hand, both Siropins are highly active at wide range of temperature (4.0–50 °C) and pH (6.0–10) indicating a certain versatility of these proteins compatible with the gastrointestinal environment.

The thermostability studies showed that Siropin 1 was fully stable at temperatures until 55 °C whereas at above temperatures its thermostability dropped rapidly: at 60 °C the enzyme’s half-life time was 10 ± 1 min. By contrast, Siropin 2 was fully stable upto 60 °C, and at 70 °C, its half-live was 21 ± 0.5 min while it was only 3 ± 0.5 min for Siropin 1 (Table 1). At higher temperatures including 80 and 90 °C the Siropin 1 was inactive but the Siropin 2 still displayed half-lives of 20 ± 2.0 min and 19 ± 0.5 min, respectively (Table 1). These results highlighted again not only the stronger thermoactivity but also the higher thermostability of Siropin 2 compared to Siropin 1. In previous reports, the serpins from the thermophilic *Clostridium thermocellum* and *Thermobifida fusca* displayed half-lives of 30 min at 70 °C and 28 min at 60 °C, respectively [9, 12].

**Table 1:** Study of the thermal stability of Siropins

	60 °C	70 °C Half-lives	80 °C (t_1/2_, min)	90 °C
Siropin 1	10 ± 1.0	3.0 ± 0.5	ND	ND
Siropin 2	>120	21 ± 1.5	20 ± 2.0	19 ± 0.5

Results are the mean and standard deviation from three independent experiments

*ND* not detected

Study of the pH stability revealed that both Siropins were highly stable at pH 7.0 and 8.0 during 8 h. At pH 6.0 the Siropin 1 was completely stable after 8 h; yet Siropin 2 displays half-live of 2.6 h. The same behavior was observed at higher pH values thus underlying the better pH stability of Siropin 1 compared to Siropin 2 (Table 2).

**Table 2:** Study of the thermal pH stability of Siropins

	5	6	7 Half-lives	8 (t_1/2_, min)	9	10
Siropin 1	210 ± 5.0	>480	>480	>480	>480	315 ± 3.0
Siropin 2	18 ± 2.0	160 ± 1.0	>480	>480	230 ± 3.0	114 ± 2.0

Results are the mean and standard deviation from three independent experiments

These different thermal and pH stability behaviors of Siropin 1 and 2 can be related to their low amino-acid sequence identity (63%). Studies on structure–function relationships of these bacterial serpin, mainly through the determination of the 3D structure of these proteins, will provide further structural explanations.

The different Siropins behaviors in terms of pH and temperature activities and stabilities can be related to the adaptation of this commensal bacterium *E. siraeum* to different ecological niches such as the stomach (acid pH) and the bowels (neutral pH).

### Kinetic studies

**Fig. 6:**
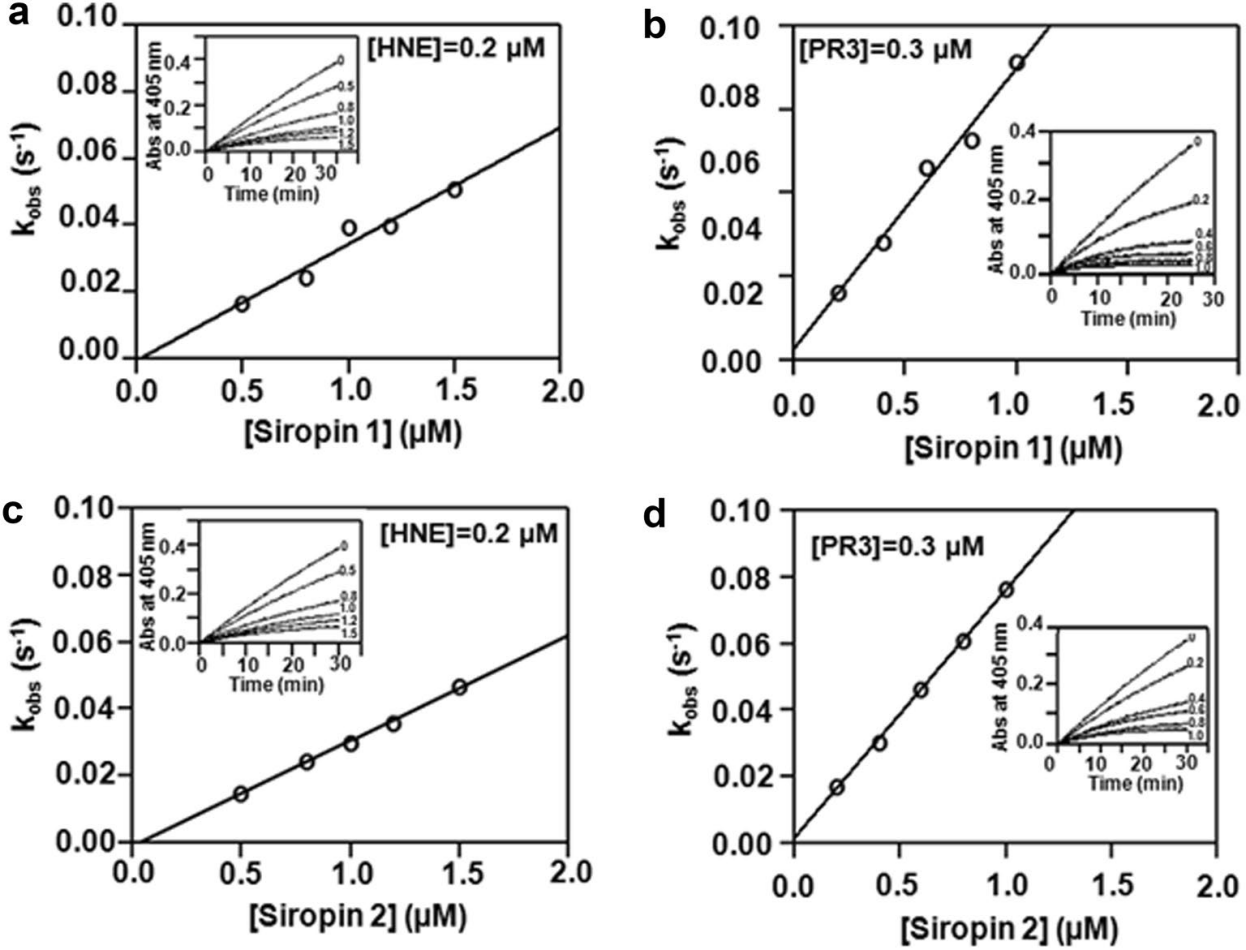
Progress curves for proteases inhibition by Siropins. **a** and **c** HNE inhibition by Siropin 1 (**a**) and Siropin 2 (**c**). **b** and **d** PR3 inhibition by Siropin 1 (**b**) and Siropin 2 (**d**). Constant concentrations of HNE or PR3 were used with various amount of Siropin, the change in the absorbance was then performed at 37 °C. Used concentration of Siropin (µM) are shown beside each progress curve. Obtained *k*_*obs*_ rate were plotted against the Siropin concentration. The apparent second-order association constant *k’*_*app*_ was calculated as the slope of the fitted linear curve and then corrected to determine the second-order association constant *k*_*a*_

As previously reported serpins inhibit proteases through an irreversible mechanism, therefore the main kinetic parameter which characterizes their inhibition efficiencies is the association rate constant (k_a_). For both target proteases studied herein the second-order association rate constants were calculated by progress curve analysis (Fig. 6). Using the Siropin 1, the k_a_ values were 8.4 ± 0.07 × 10^4^ M^−1^ s^−1^ and 2.6 ± 0.02 × 10^5^ M^−1^ s^−1^ for HNE and PR3, respectively. In case of Siropin 2 we obtained k_a_ of 7.7 ± 0.02 × 10^4^ M^−1^ s^−1^ for HNE and of 1.1 ± 0.04 × 10^5^ M^−1^ s^−1^ for PR3. These data prove that Siropins are more efficient with PR3 than with HNE. The analysis of k_a_ values of previously reported serpins towards HNE shows that serpins from bacterial origin displayed comparable values to those of Siropins (Table 3). In comparison, the eukaryotic α-1-antitrypsin and Elafin were more efficient with k_a_ values of 6.5 10^7^ and 3.7 × 10^6^ M^−1^ s^−1^, respectively (Table 3). This higher efficiency can be explained by the fact that both α-1-antitrypsin and Elafin are the natural human inhibitor of HNE. Such statement is reinforced with the observation that the α-1-antitrypsin and Elafin also inhibited better the human serine protease PR3 than Siropin 1 and 2. However, we report for the first time the fact that bacterial serpins can inhibit the human PR3. Previous studies showed that to ensure the control of target proteases in blood plasma, serpins with less than 100 ms inhibition life-time are required [35]. Taken together our findings, it can be assumed that Siropins could perform an effective protease inhibition in the gastro-intestinal tract.

**Table 3:** Association rate constant values of previously reported serpins

	HNE	PR3	Reference
Siropin 1	8.4 ± 0.07 × 10^4^	2.6 ± 0.02 × 10^5^	This study
Siropin 2	7.7 ± 0.02 × 10^4^	1.1 ± 0.04 × 10^5^	This study
α-anti-trypsin	6.5 ± 4.0 × 10^7^	1.0 ± 0.2 × 10^6^	[25, 33]
Elafin	3.7 ± 0.1 × 10^6^	3.3 ± 0.03 × 10^6^	[34]
Tengpin	1.3 × 10^5^	NR	[13]
Miropin	1.2 ± 0.05 × 10^5^	NR	[10]
Serpin from *B. longum*	4.7 × 10^4^	NR	[14]

*NR* not reported

Results are the mean and standard deviation from three independent experiments

### Siropins inhibit mice fecal proteases

**Fig. 7:**
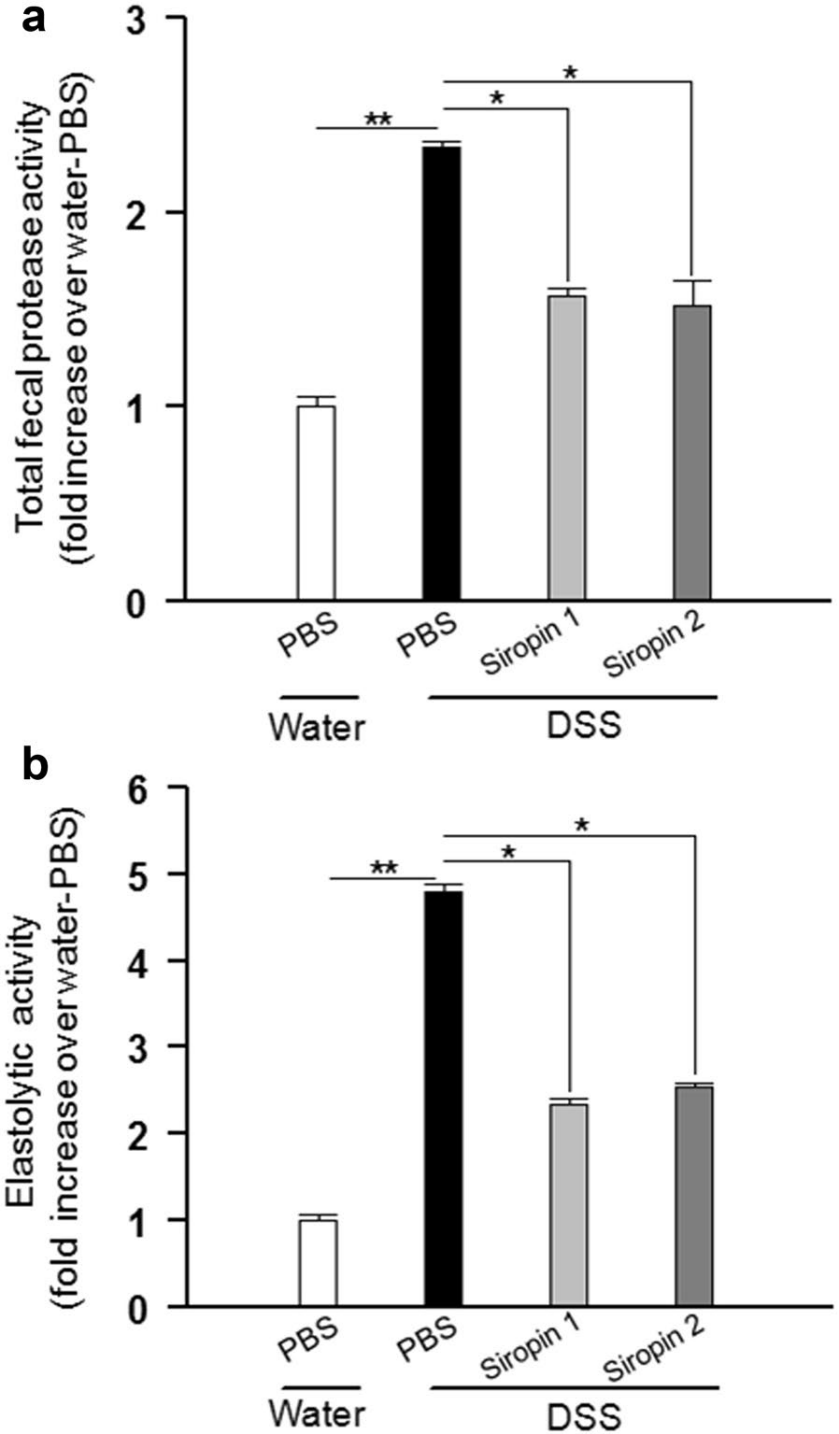
Effects of Siropin on mice fecal proteases. Inhibition of total fecal proteolytic activity (**a**) and elastolytic activity (**b**) from DSS-treated mice. Protease activities from mice drinking water alone were considered as 100%. Data are shown as mean ± SEM, *p < 0.05, **p < 0.01, ***p < 0.001 using Kruskal–Walls with Dunn’s post-test (n = 8 in each group)

To check the ability of Siropins to inhibit proteases associated with intestinal inflammation, feces were recovered from two conventional mice groups: the first group was treated with PBS while in the second group inflammation was induced with Dextran sulfate sodium (DSS, 3%). The measure of the total fecal protease activity (FPA) demonstrated that FPA in DSS-treated mice group was nearly 2.2 fold higher than that of the PBS-treated group (Fig. 7a). Such results are in accordance with those reported in case of the human IBD patients who displayed an increase of the FPA compared to that of healthy subjects [36, 37]. Hence our results confirmed again the involvement of proteases on the inflammatory bowel diseases. Several reports underlined the key role of elastolytic activity in inflammatory bowel diseases [15, 28, 29]. As shown in Fig. 7b, the level of measured elastolytic activity in mice treated with DSS was nearly fivefold higher than that of the PBS-treated mice group proving that the DSS treatment induced an increase of the protease level, and notably the elastolytic activity.

The analysis of the FPA in the presence of Siropins showed a significant decrease of the total proteolytic activity which was evaluated to 30 and 35% with Siropin 1 and 2, respectively (Fig. 7a). Interestingly when the Siropins were added, the elastolytic activity was reduced to 50% with Siropin 1 and to 46% with Siropin 2. Altogether these data establish that the addition of Siropins allowed the decrease of the FPA activity in fecal water of DSS-inflamed mice with a pronounced effect on the elastolytic activities indicating that Siropins are highly active in such conditions.

These data highlighted the efficiency of the Siropins to inhibit fecal water proteases and thus their potential as good candidates to inhibit proteases associated with inflammation. Henceforth Siropins can constitute a promising therapeutic strategy to treat inflammatory diseases.

## Conclusions

The human gut bacterium *E. sireaum* encoded two putative serpins which display low sequence identity together and with other bacterial serpins. Activity studies of these putative serpins demonstrated that they act as a serine protease inhibitors. The analysis of these novel bacterial serpins, called Siropins, revealed that they efficiently inhibit the human serine proteases HNE and PR3. Interestingly, Siropins are the first bacterial serpins that significantly inhibit the human PR3. The biochemical characterization of both Siropins indicated that Siropin 1 was the most active and stable at low pH values while Siropin 2 was more thermoactive and thermostable. These different behaviors can be associated to the adaptation of the commensal bacterium to different ecological niches. Kinetic studies demonstrated that Siropins were highly efficient in comparison to other bacterial serpins including that of *B. longum* conferring thus to Siropins an interesting potential to inhibit serine proteases and specifically those associated to several human physiopathologies. To assess this hypothesis we tested the impact of Siropins on the proteases fecal activities. This study evidenced that Siropins strongly inhibit the fecal proteases and mainly the elastolytic activities. Altogether, these results highlight the high potential of Siropins, and serpin from the human gut microbiota, as a powerful therapeutic strategy to treat protease-mediated pathologies and mainly inflammatory bowel diseases.

## Methods

### Enzymes, reagents and substrates

Used human proteases: Human Neutrophil Elastase (HNE) and Human Proteinase 3 (PR3) were purchased from elastin products company (EPC, Inc). Porcine pancreatic elastase (PPE), bovine pancreatic trypsine (BPT), bovine pancreatic chymotrypsine (BPC) and subtilisin Carlsberg (Sub) were obtained from Sigma. All substrates: Meosuc AAPV-pNA (Sigma) and MCA-RPKPVE-Nval-WRK(Dnp)-NH_2_ (Bachem) were used according to the manufacturer’s instructions.

### Bacterial strains, plasmids and media

*Eubacterium siraeaum* DSM 15702 strain was grown under anaerobic conditions in Medium 110 with 15% rumen fluid. *Escherichia coli* BL21 (DE3) were used in this study as host strain. Culture of different *E. coli* strains was done in Luria–Bertani (LB) medium. These media were supplemented, when necessary, with ampicillin (50 µg/ml), kanamycin (25 µg/ml) and IPTG (isopropyl β-d-thiogalactopyranoside) at 240 µg/ml. The pDONR-221 and pDEST-17 plasmids (Promega) were used as entry and expression vectors respectively.

### DNA manipulation and PCR

Chromosomal DNA was isolated from *E. siraeum* DSM 15702 using the Wizard^®^ Genomic DNA Purification kit (Promega). Preparation of plasmid DNA and separation of fragments by agarose gel electrophoresis were performed as described by Sambrook et al. [38]. To amplify the genes encoding for Siropins, we used gene-specific and universal primer pairs in a 1∶4 ratio to generate the attachment binding sites (underlined) attb1 and attb2 in the final constructs. Based on the *E. siraeum* complete genomic sequence available in the NCBI Databank (ID: FP929044.1), we designed the specific oligonucleotide sequences F-SP1 5′GTACAAAAAAGCAGGCTTCACAGGCATCAAAGCCGCAAGCC3′ and R-SP1 5′CAAGAAAGCTGGGTCTCATATACCTGTATACACGCC3′ for Siropin 1 gene amplification and both of F-SP2 5′GTACAAAAAAGCAGGCTTCAAAAGAAGAATTTTATCAGCA3′ and R-SP2 5′CAAGAAAGCTGGGTCTTATACGCCTGTATATATGCC3′ were used to amplify the Siropin 2 gene. Furthermore, F-33 5′GGGGACAAGTTTGTACAAAAAAGCAGGCTTC3′ and R-20 5′GGGGACCACTTTGTACAAGAAAGCTGGGTC3′ were used in this study as universal primers. Polymerase chain reactions were performed using Gene Amp^®^ PCR System 9700 (Applied Biosystems). The amplification reaction contained Takara DNA polymerase amplification buffer, 20 pmol of each primer, 50 ng of DNA template, and 5 units of Takara DNA polymerase (TaKaRa). PCR cycling parameters were: 94 °C for 5 min, followed by 25 cycles of 94 °C for 30 s, 55 °C for 60 s, and 72 °C for 120 s. PCR product was purified using QIAquick Gel Extraction Kit (Qiagen^®^) by following the manufacturer’s instructions. The purified fragments were used to be cloned into the Gateway plasmid pDONR-221 and therefore subcloned in the pDEST-17 expression vector by homologous recombination using the BP and LR clonase enzymes respectively (Invitrogen^®^). After transformation in competent *E. coli* BL21 strain, numerous recombinant clones were obtained in case of each gene. Therefore sequence of two recombinant clones harboring the Siropins 1 or 2 genes under the control of the T7 promoter with six histidines at the N-terminus of the protein, were confirmed.

### Siropins expression and purification

Recombinant *E. coli* strains were grown as indicated above until the culture reached an OD_600_ of 0.6, and the culture was induced for 3 h using IPTG (240 μg/ml) followed by harvesting of the cells by centrifugation (8000 *g*, for 15 min at 4 °C). Pellets were resuspended in 20 mM Tris–HCl buffer, pH 8.0 containing 500 mM NaCl. The cell suspensions (20 ml/pellet from 1 L of culture) were incubated for 1 h on ice in the presence of 10 mM MgCl_2_ and 200 U benzonase (Novagen^®^). Cell disruption was done by sonication at 4 °C for 1 min (three cycles of 10 s pulses at amplitude of 40%) using a Vibra-CellTM 72408 Sonicator and cell debris was removed by centrifugation (16,000*g*, for 30 min at 4 °C). Purification was performed by affinity chromatography using a 1 ml HiTrap chelating HP column (GE Healthcare) charged with Ni^2+^ ions on the ÄKTA Purifier FPLC system (Amersham Pharmacia Biotech) and equilibrated with 20 mM Tris–HCl pH 8.0 buffer supplemented with 500 mM NaCl. Proteins were eluted using linear imidazole gradient ranging from 0 to 500 mM. The fractions containing serpins activity were pooled and protein purification was achieved by size exclusion chromatography using Superdex S-200 10/300 GL column (GE Healthcare). Proteins elution was done through an isocratic gradient with a flow rate of 0.3 ml/min in the same buffer.

### Protein analysis, amino acid sequence alignment and homology modeling

Protein concentration was determined by measuring the UV absorption at 280 nm using a Nanodrop device (Thermos Fisher Scientific). The protein samples were separated in 12% SDS-PAGE according to the Laemmli method [39], and bands were visualized by Coomassie brilliant blue R-250 (Bio-Rad) staining. The estimated molecular mass of purified Siropins was confirmed by the size exclusion chromatography. Correspondence of purified proteins to Siropins was confirmed by mass spectrometry and western blotting approaches.

Purified Siropins were analyzed by SDS-PAGE and then electrotransferred to nitrocellulose membranes (Hybond-ECL, Amersham Biosciences) which were blocked in phosphate-buffered saline solution containing 0.05% Tween 20 (v/v) and 5% defatted milk powder (wt/vol) at 4 °C during 1 h. Subsequently, blots were incubated with primary antibody (with 1/10,000 dilution of mouse anti-polyHistidine monoclonal antibody, Sigma) at room temperature for 1 h. Treated membranes were washed three times with the phosphate-buffered saline solution and incubated with goat anti-mouse HRP (Bio-Rad) during 1 h. Blot signals were detected using chemiluminescent substrate and ChemiDoc™ MP System (Bio-Rad).

The multiple alignment of the Serpin amino-acid sequence was done using the program ClustalW [40] and the figure rendering was done using the ESPript sequence analysis server [41]. The 3D homology model of Siropin 1 and 2 was generated using the Geno3D server, and images were rendered by using VIEWERLITE™ 5.0.

### Enzyme inhibition assays

All reactions were assayed in 20 mM tris–HCl, 500 mM NaCl, pH 8.0 buffer. All substrates were suspended in DMSO and stored at −80 °C and used in the final concentration of 200 µM for MeOSuc-AAPV-pNA and 10 µM for MCA-RPKPVE-Nval-WRK(Dnp)-NH_2_. Reaction mixture is composed of each serine protease with equimolar concentration of Siropin in the appropriate buffer and then incubated during 30 min. Inhibition is measured by addition of the chromogenic or fluorogenic substrates in a final volume of 100 µl in 96-well plates with clear or black bottom, respectively. Residual activity was determined during 20 min at 37 °C using a plate reader (Synergy™ 2 Multi-Mode Microplate Reader, BioTek^®^) for pNA substrates (absorbance at 405 nm) and (Dnp)-NH_2_ (excitation 360 nm, emission 460 nm). The final concentration of enzyme was 0.2, 0.3 and 1 µM for HNE, PR3 and PPE, respectively. Concentration of 50 nM was used for subtilisin (Sub), bovine pancreatic trypsin (BPT) and bovine pancreatic chymotrypsin (BPC).

### Stoichiometry of inhibition

Stoichiometry of inhibition was determined by varying the amount of Siropins with a concentration of 200 nM for HNE and 300 nM for PR3. In fact, proteases and Siropins were gently mixed yielding to molar ratios of protease/inhibitor ranging from 0 to 10 during 5 min at 37 °C. Therefore, substrate was then added and activity was assessed for 30 min by measuring absorbance at 405 nM. Fractional activity (velocity of the inhibited enzyme reaction/velocity of the non inhibited enzyme reaction) was subsequently calculated and plotted to the concentration ratio of the inhibitor to enzyme ([I0/E0]). The stoichiometry of inhibition was determined by linear regression as the x-intercept.

### Determination of the P1–P1′ site of siropins and mass spectrometry analysis

The RCL cleavage site was determined by mixing Siropin (40 µM) with HNE or PR3 in a total volume of 10 µl. Final enzyme/inhibitor ratio was 3 for HNE and 1 for PR3. After incubation for 5 min at 25 °C, the reaction was stopped by addition of 20 µl of boiling SDS-PAGE sample buffer followed by incubation for 5 min at 100 °C. Samples were then subjected to electrophoresis under reducing conditions according to the Laemmli method [39]. Subsequently, Protein bands of the Coomassie-stained gel were excised and subjected to mass spectroscopy. The gel pieces were washed, reduced using DTT and alkylated with iodacetamide. 20–30 µl of 3 M HCl was then added and the dehydrated gel pieces were microwaved for 10 min at 900 W. The supernatant was removed and desalted directly on Oasis HLB Elution Plate (Waters). Samples were eluted in 50 μl, dried in a speed vacuum centrifuge and dissolved in 10 μl of reconstitution buffer (96:4 water/acetonitrile and 0.1% formic acid) and analyzed by LC–MS/MS. For this analysis, peptides were separated using the nanoAcquity UPLC system (Waters) fitted with a trapping (nanoAcquity Symmetry C_18_, 5 μm, 180 μm × 20 mm) and analytical column (nanoAcquity BEH C_18_, 1.7 μm, 75 μm × 200 mm) coupled directly to an linear trap quadropole (LTQ) OrbitrapVelos (Thermo Fisher Scientific) with a Proxeonnanospray source. The peptides were introduced into the mass spectrometer (OrbitrapVelos Pro, Thermo) and a spray of 2.2 kV was applied. Peptides analysis was performed using MaxQuant Software (version 1.0.13.13). The data were searched against a species-specific (*E. siraeum*) Uniprot database with a list of common contaminants appended.

### Temperature, pH and stability profiles

The effect of temperature on the serpin activity was determined by incubating of each purified Siropin for 30 min at various temperatures ranging from 4 to 90 °C and then the measurement of the residual inhibition was monitored. The Siropins pH profiles were obtained at different pH values between 2 and 10 (using 20 mM Tris–HCl, 500 mM NaCl). The stability as a function of temperature and pH was carried out by incubating each Siropin at different temperature and pH during different period and measuring residual activity.

### Kinetic characterization of siropins

Kinetics of HNE and PR3 inhibition by Siropins were studied by the progress curve method [42, 43] under pseudo-first-order conditions, where the initial concentration of Siropin were varied from 1 to tenfold greater than that of proteases. Initial reaction mixture contains substrate at final concentration of 200 µM and various amount of Siropins incubated at 37 °C for 10 min. Thereafter 10 µl of HNE (200 nM) or PR3 (300 nM) were added to the mixture and the rate of the substrate hydrolysis was monitored at 405 nm for 20 min. The progress curves were fitted by non linear regression and the pseudo first order association rate constant, *k*_*obs*_, was calculated using the Eq. 1 [42, 43].

1 $$ P = {{\left( {{{v_{0} } \mathord{\left/ {\vphantom {{v_{0} } {k_{obs} }}} \right. \kern-0pt} {k_{obs} }}} \right)} \mathord{\left/ {\vphantom {{\left( {{{v_{0} } \mathord{\left/ {\vphantom {{v_{0} } {k_{obs} }}} \right. \kern-0pt} {k_{obs} }}} \right)} {1 - e^{{ - t.k_{obs} }} }}} \right. \kern-0pt} {1 - e^{{ - t.k_{obs} }} }} $$

where *P* is the product concentration, *v*_*0*_ is the substrate hydrolysis velocity and t is the reaction time. Determined *k*_*obs*_ were plotted as a function of Siropin concentration. The apparent second order associate constant *k′*_*app*_ was considered to be the slope of the fitted linear curve. Taking into account the competitive nature of inhibition by serpin, the second order association rates were then corrected using the Eq. 2 [42, 43].

2 $$ K_{a} = k_{app}^{'} \left( {{{1 + \left[ S \right]} \mathord{\left/ {\vphantom {{1 + \left[ S \right]} {K_{m} }}} \right. \kern-0pt} {K_{m} }}} \right) $$

where [*S*] is substrate concentration and *K*_*m*_ is the Michaelis–Menten constant determined from the Lineweaver–Burk plot. *K*_*m*_ values were 114 µM for HNE and 270 µM for PR3.

### Fecal protein extraction and inhibition analysis

Fecal samples collected from 57BL/6 J (6–8 weeks old) treated with phosphate buffer and DSS dissolved in drinking water (3%) during 7 days with free access to food and water. All procedures were performed according to the European Community Rules and approved by the Animal Care Committee (C2E−45 COMETHEA) with authorization number A78-322-6.

Feces were homogenized in cooled 20 mM Tris–HCl, 150 mM NaCl, pH8. Cells were subsequently lyzed by sonication at 4 °C for 1 min (3 cycles of 10 s pulses at amplitude of 40%) using a Vibra-CellTM 72408 Sonicator and cell debris was removed by centrifugation (15,000*g*, for 30 min at 4 °C). Recovered supernatants containing extracted proteins were then filtered (0.45 µm) and used for monitoring the fecal protease activity.

Reactions were performed in 20 mM Tris–HCl, 150 mM NaCl, pH 8. Inhibition of total fecal proteolytic activity was performed in 1 ml reaction volume containing fecal proteins (0.1 mg/ml) with various amounts of Siropins and 100 µl of casein 1% (w/v). After incubation during 1 h at 37 °C, casein hydrolysis was stopped with 500 µl of 20% (w/v) trichloracetic acid (sigma). Precipitated proteins were removed by centrifugation (15,000*g*, 15 min at 4 °C) and the residual enzymatic activity was measured in the clear supernatant at 280 nm.

The effect of Siropin on elastolytic activities were measured in 100 µl reaction volume using MeOSuc-AAPV-pNA as substrate. Each fecal sample (4 mg/ml) was incubated with different amount of Siropin and then incubated for 30 min at 25 °C. Subsequently, substrate (150 µM) was added to the mixture and the absorbance change was monitored at 405 nm over 30 min at 37 °C using a microplate reader (Synergy™ 2 Multi-Mode Microplate Reader, BioTek^®^).

## Additional file

**Additional file 1: Figure S1.** The predicted 3-dimensional structure of Siropin 1 and 2 showing the reactive center loop and the N-termini highlighted in red.
