# Supplementary material for: Siropins, novel serine protease inhibitors from gut microbiota acting on human proteases involved in inflammatory bowel diseases
Source: Microb Cell Fact. 2016 Nov 29;15:201. doi: 10.1186/s12934-016-0596-2 (PMC5127057; doi:10.1186/s12934-016-0596-2)
Supplement: Supplementary file 1 — Additional file 1: Figure S1. The predicted 3-dimensional structure of Siropin 1 and 2 showing the reactive center loop and the N-termini highlighted in red. [file 12934_2016_596_MOESM1_ESM.docx]

**Siropins, novel serine protease inhibitors from gut microbiota acting on human proteases involved in inflammatory bowel diseases**

**Additional file 1**

Héla Mkaouar^1,2^, Nizar Akermi^1,2^, Vincent Mariaule^3^, Samira Boudebbouze^1^, Nadia Gaci^1^, Florette Szukala^1^, Nicolas Pons^4^, Josan Marquez^3^, Ali Gargouri^2^, Emmanuelle Maguin^1^, and Moez Rhimi^1^*

^1^ UMR 1319 Micalis, INRA, AgroParisTech, Université Paris-Saclay, F-78350 Jouy-en-Josas, France.

^2^ Laboratory of Molecular Biology of Eukaryotes, Center of Biotechnology of Sfax, University of Sfax, 3038 Sfax, Tunisia.

^3^ European Molecular Biology Laboratory, Grenoble Outstation, 71 Avenue des Martyrs, CS 90181, 38042 Grenoble Cedex 9, France.

^4^ INRA, Institut National de la Recherche Agronomique, US 1367 Metagenopolis, Jouy-en-Josas, France

***** Corresponding authors:

Moez Rhimi

*E-mail address*: moez.rhimi@jouy.inra.fr

Tel. +33 1 34 65 22 94 / Fax: +33 1 34 65 25 21

**Figure S1.** The predicted 3-dimensional structure of Siropin 1 and 2 showing the reactive center loop and the N-termini highlighted in red.
